# Computational Insight Into the Structural Organization of Full-Length Toll-Like Receptor 4 Dimer in a Model Phospholipid Bilayer

**DOI:** 10.3389/fimmu.2018.00489

**Published:** 2018-03-12

**Authors:** Mahesh Chandra Patra, Hyuk-Kwon Kwon, Maria Batool, Sangdun Choi

**Affiliations:** ^1^Department of Molecular Science and Technology, Ajou University, Suwon, South Korea; ^2^Department of Orthopaedics and Rehabilitation, Yale School of Medicine, New Haven, CT, United States

**Keywords:** full-length TLR, TLR4, plasma membrane, molecular dynamics simulation, signal transduction, adaptor recruitment

## Abstract

Toll-like receptors (TLRs) are a unique category of pattern recognition receptors that recognize distinct pathogenic components, often utilizing the same set of downstream adaptors. Specific molecular features of extracellular, transmembrane (TM), and cytoplasmic domains of TLRs are crucial for coordinating the complex, innate immune signaling pathway. Here, we constructed a full-length structural model of TLR4—a widely studied member of the interleukin-1 receptor/TLR superfamily—using homology modeling, protein–protein docking, and molecular dynamics simulations to understand the differential domain organization of TLR4 in a membrane-aqueous environment. Results showed that each functional domain of the membrane-bound TLR4 displayed several structural transitions that are biophysically essential for plasma membrane integration. Specifically, the extracellular and cytoplasmic domains were partially immersed in the upper and lower leaflets of the membrane bilayer. Meanwhile, TM domains tilted considerably to overcome the hydrophobic mismatch with the bilayer core. Our analysis indicates an alternate dimerization or a potential oligomerization interface of TLR4-TM. Moreover, the helical properties of an isolated TM dimer partly agree with that of the full-length receptor. Furthermore, membrane-absorbed or solvent-exposed surfaces of the toll/interleukin-1 receptor domain are consistent with previous X-ray crystallography and biochemical studies. Collectively, we provided a complete structural model of membrane-bound TLR4 that strengthens our current understanding of the complex mechanism of receptor activation and adaptor recruitment in the innate immune signaling pathway.

## Introduction

Toll-like receptors (TLRs) are key components of the vertebrate innate immune system and play a dominant role in the activation of the adaptive immune system (1–3). They are pattern recognition receptors that recognize exogenous pathogen-associated molecular patterns or endogenous damage-associated molecular patterns to initiate a complex cascade of signal transduction to produce pro-inflammatory cytokines and interferons (IFNs) (4). TLRs are found in the plasma membrane (TLR1, 2, 4, 5, 6, and 10) as well as in the endosomal membrane (TLR3, 7, 8, and 9), recognizing distinct categories of ligands (5). Specifically, triacyl lipopeptides (Pam_3_CSK_4_) are recognized by TLR1/2 (6), diacyl lipopeptides (Pam_2_CSK_4_) by TLR2/6 (7), viral double-stranded RNA by TLR3 (8, 9), lipopolysaccharides (LPS) by TLR4 (10–12), bacterial flagellin by TLR5 (13), viral single-stranded RNA by TLR7 and TLR8 (14, 15), and bacterial CpG-containing DNA by TLR9 (16). Ligands recognized by TLR10 are unknown; however, evidence indicates that TLR10 can recognize viral or bacterial components (17, 18).

Ligand-induced TLR dimerization results in the recruitment of the downstream adaptor, myeloid differentiation primary response gene 88 (MyD88), or in the case of TLR3, toll/interleukin-1 receptor (TIR) domain-containing adapter-inducing interferon β (TRIF). TLR4 also initiates TRIF-dependent immune signaling from the endosomal membrane. Therefore, TLR4 is most unique one among TLRs, as it can trigger LPS-induced MyD88- and TRIF-dependent signal transduction from both the plasma membrane and endosomal membrane, respectively. In the MyD88 pathway, activated TLRs recruit MyD88 through TIR domain interactions. MyD88, in turn, recruits the interleukin-1 receptor-associated kinase 4 (IRAK4) through death domain interactions. IRAK4 phosphorylates IRAK1, which brings tumor necrosis factor receptor-associated factor 6 to the receptor complex (19). The TLR-MyD88-IRAK4-IRAK1/2 supercomplex is termed myddosome, whose actual stoichiometry is still under debate (20, 21). Subsequently, a number of phosphorylation and ubiquitination events occur that eventually activate nuclear factor kappa-light-chain-enhancer of activated B cells (NF-κB). Similarly, the TRIF-dependent pathway employs a different set of adaptors and kinases to activate IFN regulatory factor 3 (IRF3)—a transcription factor. Activated NF-κB and IRF3 translocate to the nucleus and assist in the transcription of pro-inflammatory cytokines: interleukin-1 (IL-1), IL-6, IL-10, IFN1β, and IFNγ (22).

Structurally, TLRs show a tripartite domain architecture with an extracellular ligand binding domain (ECD) containing leucine-rich repeats (LRR), a single transmembrane (TM) domain, and an intracellular TIR domain (ICD). Agonist binding induces homo- or heterodimerization of TLRs that laterally translocate in the membrane until the recruitment of downstream adaptors. TLR4-ECD is tightly associated with a co-receptor, myeloid differentiation protein 2 (MD2), which traps agonists (such as LPS) in its large hydrophobic cavity and plays a significant role in the activation of the receptor. Conformational changes in TIR domains provide a platform for adaptors that then propagate signal transduction. Several studies have been conducted to uncover the three-dimensional structures of proteins involved in this complex pathway. TLR structural biology has been reviewed in detail elsewhere (23). The extracellular domains of all TLRs (6, 7, 9, 13, 16, 24–26), except TLR10, and the TIR domains of some TLRs (27–29) have been solved through X-ray crystallography. The TM domains of TLR3 (30) and TLR4 (31) have been recently solved through NMR spectroscopy, suggesting a hypothetical model for full-length TLR4. However, experimental or computational studies elucidating the full-length structure of TLRs with an intact ECD-TM-ICD organization have not been reported so far. A complete understanding of full-length TLRs would aid identification of receptor micro-domains that participate in membrane association and orientation of individual domains in physiological environments. Such regions could be targeted using novel activators or inhibitors for modulating different functional properties of TLRs to obtain the desired therapeutic outcomes (32–38).

In this study, we predicted the putative structural organization of full-length TLR4 in a membrane-mimetic environment. The prediction provided several key insights into the orientation and interaction of ECD, TM, and TIR domains with respect to the membrane bilayer. Since these domains are independent and well-validated drug-targets, detailed understanding of their interactions with the plasma membrane and dimeric counterparts is a prerequisite for the development of peptide- or small-molecule-based therapeutics.

## Materials and Methods

### Construction of a Full-Length TLR4-MD2-LPS Homo-Heterodimer and Individual TIR and TM Homodimers

The construction of a full-length TLR4 dimer was completed in five successive stages. First, the dimeric LPS-bound ECD structure was obtained from the protein data bank (PDB ID: 3FXI). Missing residues were modeled *via* homology modeling using the SWISS-MODEL server (39), followed by energy minimization using GROMACS software version 5.1.4 (40). Second, the TM domain (residues 630–660) was modeled as a single α-helix, followed by protein–protein docking using the ZDOCK server (41) to obtain a dimeric structure. Energy minimization was performed to optimize interatomic distances and angles. Third, the TIR domain was modeled by homology modeling using the crystal structure of TLR10 (PDB ID: 2J67) that was solved in physiological dimeric conditions (28). Consecutive superimposition of monomeric TLR4-TIR over the two subunits of dimeric TLR10-TIR resulted in a dimeric TLR4-TIR domain. Energy minimization was performed to remove steric conflicts between atoms. Fourth, all three individual domains were aligned on a straight axis and peptide bonds were patched between the extreme C- and N-terminal residues of adjacent domains using Discovery Studio Visualizer 4.0 (DSV 4.0) program (Dassault Systèmes, San Diego, CA, USA). Another round of energy minimization was performed to correct interatomic conflicts within the full-length TLR4 dimer. Finally, the residues around the constructed peptide bonds were optimized using the ModLoop webserver (42).

In addition, individual TIR and TM dimers were constructed using the protein–protein docking approach. For TLR4-TIR, two different models were created based on the dimer packing information available in the literature (28, 37, 43, 44). The first TIR dimer was created by successive superimposition of TLR4-TIR monomers over those of the dimeric TLR10-TIR. The second TIR dimer was obtained by performing protein–protein docking using the BB loop of one subunit and the helix αE of the other as binding regions. The TM domain dimer was constructed using an automated protein–protein docking approach with the ZDOCK program. The best scoring predictions were selected for further study.

### Construction of Lipid Bilayers and Insertion of TLR4 Into the Bilayer

TLR4 was simulated in two separate dipalmitoylphosphatidylcholine (DPPC) bilayers with 574 lipids to observe if dynamic properties of both TLR4 and the membrane are replicated. Initially, a pre-equilibrated lipid bilayer of 128 DPPC molecules was obtained from the Peter Tieleman website.^1^ The bilayer was replicated in *X* and *Y* directions using the GROMACS *gmx conf* tool to accommodate TLR4-ECD in lateral directions. The resultant bilayer was energy minimized and simulated for 100 ns. TLR4 was inserted inside the DPPC bilayer by aligning the hydrophobic segments of TLR4-TM with that of the membrane. InflateGRO methodology was used for the packing of lipids around TLR4 (45).

### Molecular Dynamics (MD) Simulation Parameters for Modeled TLR4 Dimers in Phospholipid Bilayers

A hybrid force field was created by combining Gromos96-54a7 and Berger-lipid parameters for simulating the TLR4-MD2-LPS system. An appropriate amount of simple point charge (SPC) water molecules and counterions (Na^+^/Cl^−^) were added to the simulation system. Energy minimization was carried out using the steepest descent algorithm in GROMACS. Temperature and pressure couplings were performed for 100 ps each using Nose–Hoover and Parrinello–Rahman methods, respectively, with positional restraints on the backbone heavy atoms. The production run was carried out for 100 ns using the NPT ensemble (constant pressure, constant temperature) without backbone restraints. Short-range van der Waals and electrostatic interactions were calculated using a 12 Å distance cutoff. Long-range electrostatic interactions were handled using the particle mesh Ewald method. Periodic boundary condition was applied to the simulation system and all bonds were constrained using the linear constraint solver algorithm. Structural snapshots were saved at 2 ps time intervals. Trajectory data analysis was performed using visual molecular dynamics (VMD) (46), PyMOL (Schrödinger, LLC, New York, NY, USA), DSV 4.0, XMgrace,^2^ and built-in GROMACS tools. LPS topology was computed using the automated topology builder server (47) which uses a hybrid quantum mechanics/molecular mechanics method for assigning partial charges to atoms. The volume of the MD2 hydrophobic cavity was computed using *trj_cavity_v2.0* program (48).

### MD Simulation of Isolated TLR4-TIR Dimers

Two separate MD simulations were performed for the isolated TLR4-TIR dimers in two different dimeric orientations, as reported in the literature (37, 43, 44, 49). The models were solvated with SPC water molecules inside separate cubic boxes. Counterions (Na^+^/Cl^−^) were added and energy minimization was performed using Gromos96-54a7 force field and steepest descent algorithm. Temperature and pressure equilibrations were carried out using V-rescale and Parrinello-Rahman coupling schemes, respectively. Remainder of the parameters were the same as described for TLR4-membrane simulations.

### MD Simulation of an Isolated TLR4-TM Dimer

A separate MD simulation was carried out for the isolated TM segment of TLR4 (residues 630–660) in a pre-equilibrated DPPC bilayer. The dimeric TM domain was placed inside the hydrophobic core of a DPPC membrane. The simulation was performed for 100 ns using the same set of parameters described for TLR4 in the previous section.

### Electrostatic Potential Calculation

The molecular electrostatic potential surfaces were calculated using the adaptive Poisson–Boltzmann solver and PyMOL *apbsplugin* tool.^3^ The solvent accessible surface area was computed using a linearized Poisson–Boltzmann equation with a bulk solvent radius of 1.4 Å. The isosurfaces (positive and negative spheres) were viewed using a contour (kT/e) value of 1.

### Free Energy Landscape (FEL)

The FEL was calculated to obtain lowest energy conformations of the modeled TLR4, which were then evaluated for stereo-chemical accuracy using ProSA-Web (50) and the Rampage servers (51). The FEL was calculated using the GROMACS *gmx sham* tool and the landscape was plotted using the demo version of Mathematica software (version 11.2; Wolfram Research, Inc., Champaign, IL, USA).

### Molecular Docking of the TAK-242 Ligand With TLR4-TIR Dimers

The chemical structure of TAK-242 was obtained from the PubChem database (CID: 11703255). Then, the structure was protonated and energy minimized using the molecular operating environment program (52). The binding site was defined around the C747 residue and docking was performed using the London-DG scoring function and MMFF94x force field optimization. A total of 30 different docked conformations were generated and the best pose was selected based on the binding affinity (*S*) score.

### Binding Free Energy Calculation of TIR Dimer and TIR-TAK-242 Complexes

The molecular mechanics/Poisson–Boltzmann surface area (MM/PBSA) method (53) was employed to calculate the binding free energies between different components. The method is summarized by Eq. 1,

(1) $$\Delta G_{\text{bind}}=\langle \Delta G_{\text{complex}}\rangle -\langle \Delta G_{\text{protein}}\rangle -\langle \Delta G_{\text{ligand}}\rangle$$

where *G*_bind_ denotes the binding free energy and *G*_complex_, *G*_protein_, and *G*_ligand_ represent the free energy of individual states. The free energy of each state is calculated by Eq. 2,

(2) $$G=G_{\text{bond}}+G_{\text{ele}}+G_{\text{vdW}}+G_{\text{pol}}+G_{\text{npol}}-\text{TS}$$

where *G*_bond_ (bonded, angle, and dihedral), *G*_ele_, and *G*_vdW_ are bonded, electrostatic, and van der Waals interaction energies derived from molecular mechanics energy calculations, respectively. *G*_pol_ and *G*_npol_ represent the polar and nonpolar solvation energies obtained by solving the Poisson–Boltzmann equation and solvent accessible surface area methods. The entropic contribution, TS (absolute temperature T multiplied by entropy S), is generally estimated by normal mode analysis. However, the *g_mmpbsa* program (54, 55), which we used for binding free energy calculation, ignores entropic contribution to improve computational efficiency. In computational binding free energy calculations, the computation of the entropic term often overestimates obtained binding free energy, resulting in misleading outcomes (56).

## Results

### The Full-Length TLR4-MD2 Complex Tilts and Wraps Over the Membrane Surface

**Figure 1:**
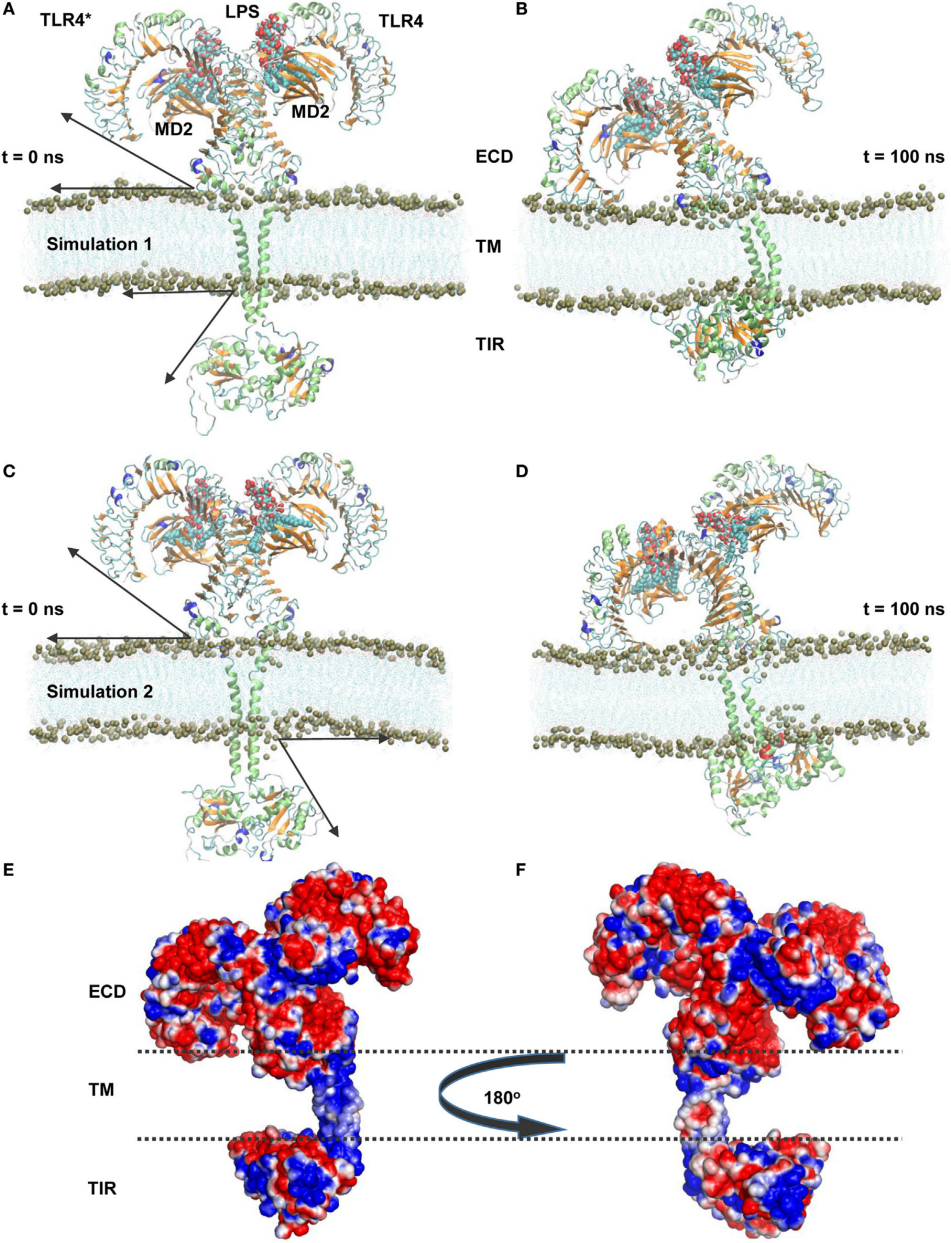
Overall structural organization of full-length TLR4-myeloid differentiation protein 2 (MD2)-lipopolysaccharides (LPS) homo-heterodimers in a membrane-embedded condition. **(A,C)** Initial models of the TLR4-MD2-LPS complex in a dipalmitoylphosphatidylcholine membrane. **(B,D)** Final snapshots of the TLR4-MD2-LPS complex after 100 ns of molecular dynamics simulation. **(A,B)** represent simulation 1, while **(C,D)** represent simulation 2. For TLR4 and MD2, the lime color represents the α-helix, orange color represents the β sheets, and LPS is illustrated as a calotte model. Arrows indicate the approximate distance traveled by extracellular and intracellular domains above and below the membrane from their starting positions. Phospholipids are indicated by lines, while phosphorous (P8) atoms are represented by mauve beads. **(E,F)** Electrostatic potential surface around the TLR4-MD2-LPS complex. The transmembrane region is marked by dashed lines.

To understand the structural organization of an intact TLR4, we performed two separate MD simulations of a full-length TLR4-MD2-LPS homo-heterodimer (residues 24–839) solvated inside a phospholipid bilayer for 100 ns durations (Figure 1). We observed that TLR4 experienced a significant rotation and structural transition in the membrane bilayer (simulation 1). The ECD progressively became inclined over the membrane to the left of the bilayer normal (*Z*-axis) and ultimately placed its N-terminal subdomain (LRR-NT) and LRR1-3 into the polar headgroups of the bilayer surface (Figures 1A,B). Meanwhile, the TM helices exhibited a substantial orientation that tilted with respect to the average helical axes due to hydrophobic mismatch with the bilayer core. Likewise, the TIR domains gradually moved upward and were partially immersed in the lower leaflet of the bilayer. In the final MD simulation snapshot, TLR4 was found completely wrapped around both upper and lower surfaces of the membrane in a slanted manner (Figure 1B). To confirm the dynamic behavior of TLR4 within the membrane bilayer, we repeated the MD simulation with a marginally upward (~2 Å) placement of the TM helices in the bilayer (simulation 2). TLR4-ECD of simulation 2 showed a similar behavior to simulation 1 by inclining over the membrane, where the LRR-NT was completely buried inside the phospholipid headgroups (Figures 1C,D). The TM helices were comparatively linear with respect to the bilayer normal; however, the first 10 residues of subunit A lost helicity. The helical tilt angle was visibly smaller than that of TLR4 in simulation 1, indicating that the placement of TM helices in simulation 1 was more precise. Finally, the TIR domain was partially absorbed into the bilayer lower leaflet, but slightly to the right of the *Z*-axis.

### The TLR4-MD2 Homo-Heterodimer Is Stable in the Phospholipid Bilayer During MD Simulation

**Figure 2:**
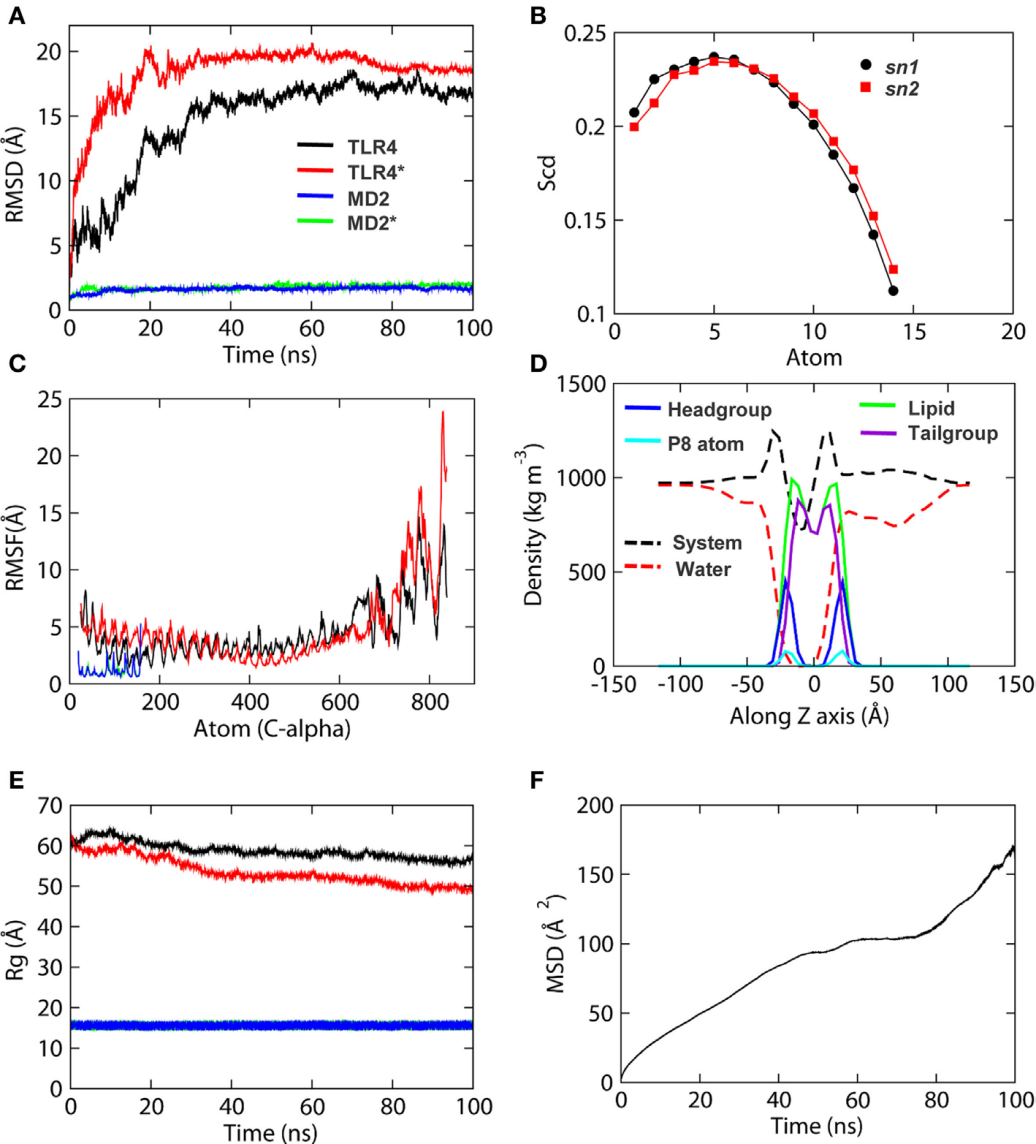
Stability parameters of the TLR4-myeloid differentiation protein 2 (MD2) complex and dipalmitoylphosphatidylcholine (DPPC) membrane of simulation 1 as a function of time. **(A)** Root mean square deviation. **(B)** Order parameters for lipid acyl chains. **(C)** Root mean square fluctuation. **(D)** Density profiles of various components of the membrane. **(E)** Radius of gyration. **(F)** Lateral diffusion of lipid head groups, also known as mean square displacement (MSD) of lipids. MSD values are based on diffusion of DPPC headgroup P8 atoms. “*” indicates chain B of TLR4 and MD2.

Before studying the detailed structural properties of membrane-bound TLR4-MD2-LPS homo-heterodimers, we checked the stability of both protein subunits and phospholipids as a function of simulation time. The root mean square deviation (RMSD) of the backbone (N-Cα-C) atoms indicated that both TLR4 chains reached an equilibrium plateau shortly after ~40 ns of MD simulation (Figure 2A). The backbone RMSD of MD2 showed an exceptional stability oscillating around ~2 Å throughout the simulation. The root mean square fluctuation (RMSF) of Cα atoms showed that the TIR domain residues were highly flexible throughout the simulation, in that the C-terminal residues of TLR4* (chain B) reached an RMSF higher than 20 Å (Figure 2C). On the other hand, local fluctuation of MD2 residues was largely restricted, indicating a stable molecular structure. Similarly, the radius of gyration (Rg) values indicated that MD2 maintained a compact architecture throughout the simulation. However, TLR4 showed an elevated Rg value of 50 Å toward the end of MD simulation, probably due to its extended molecular geometry spanning through the membrane (Figure 2E). The secondary structures of individual subunits of the TLR4-MD2 dimeric complex were largely conserved during MD simulation.

The quality of our TLR4 model was validated by calculating the Φ and Ψ dihedral angles using the Rampage server. For this task, a representative low energy structure was extracted from the Gibbs FEL (Figure S1A in Supplementary Material) using the *get_timestamp.py* script.^4^ The Ramachandran plot showed that >90% of both TLR4 and TLR4* residues fell under the most favorable and allowed regions of the plot (Figures S1C,D in Supplementary Material; Table 1). A few residues located in the flexible loops were found to have outlier dihedrals. The *Z*-score obtained from the ProSA-web server indicated that both subunits of TLR4 occupy a region defined for X-ray crystallographic structures (Figures S1E,F in Supplementary Material).

**Table 1:** Model validation scores of representative TLR4 models from two separate molecular dynamics simulations.

Subunit	Ramachandran plot^a^			ProSA-web *Z*-score
	Favored region	Allowed region	Outliers region	
**Simulation 1**				
TLR4	726 (89.2%)	64 (7.9%)	24 (2.9%)	−7.25
TLR4*	730 (89.7%)	62 (7.2%)	22 (2.7%)	−7.99
**Simulation 2**				
TLR4	719 (88.3%)	76 (9.3%)	19 (2.3%)	−7.01
TLR4*	720 (88.5%)	80 (9.8%)	14 (1.7%)	−7.07

*^a^Distribution of non-glycine and non-proline amino acids in the Ramachandran plot*.

*Indicates subunit B of TLR4.

*% represents total number of non-glycine and non-proline residues in the protein*.

Next, we analyzed key biophysical properties of the membrane bilayer to confirm its consistency throughout MD simulations. Density profiles of various membrane components revealed low and high densities at the hydrophobic and hydrophilic regions, respectively (Figure 2D). Water density at the hydrophobic core (*Z* = 0) of the bilayer was 0. The distance between headgroup densities was 37–38 Å, which is the approximate thickness of the bilayer (57). The density of tailgroups was higher than that of headgroups. Phosphorous atom (P8) density showed a good correlation with that of the headgroups. Altogether, the symmetric density of the lipids indicated a well-organized bilayer holding the dimeric TLR4. The area per lipid (A/L) of both top and bottom leaflets of the bilayer were calculated to be 58.2 ± 1, consistent with previous simulations of DPPC membranes and experimental A/L values for DPPC bilayers (58–60). Order parameters (−S_CD_) of the *sn1* and *sn2* chains of DPPC lipids showed a plateau at ~0.2 for carbon atoms 34–39 and 17–22, respectively (Figure 2B), consistent with experimental measurements (58, 61). The mean square displacement plot of lipids (lateral diffusion) exhibited a linear curve, indicating that phospholipid movements within the bilayer were natural (Figure 2F). The stability and stereochemical parameters of TLR4-DPPC system of simulation 2 are shown in Table 1, Figures S1 and S2 in Supplementary Material. Altogether, we concluded that the modeled, full-length TLR4 can be considered for further in-depth structural analysis.

### The ECD Conserves Key Molecular Features While Inclining Over the Membrane

**Figure 3:**
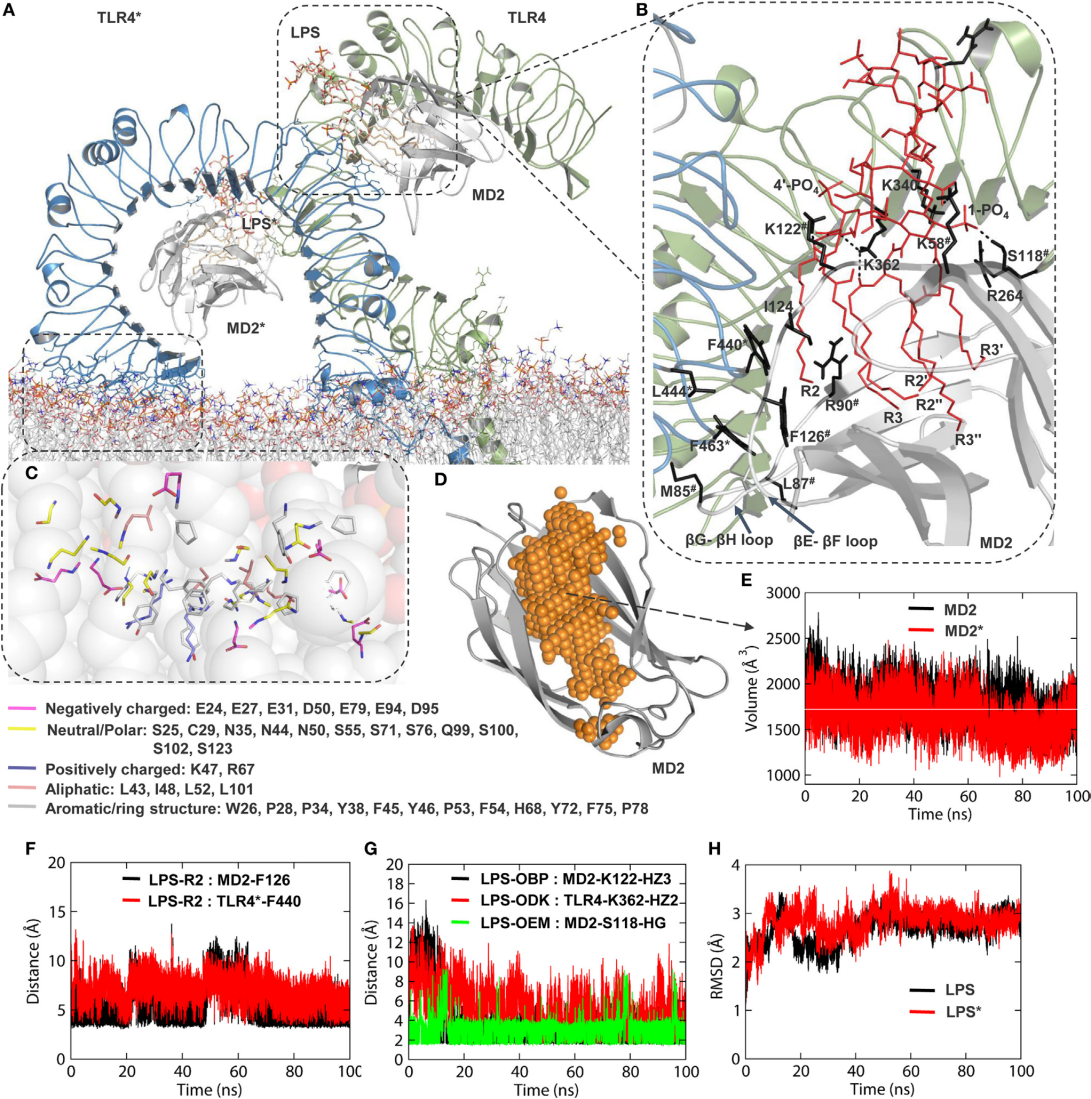
Structural properties of TLR4-extracellular ligand binding domain (ECD) and myeloid differentiation protein 2 (MD2) during molecular dynamics simulation. **(A)** Inclination of ECD over the membrane surface, as seen in the representative low energy model obtained at 94.702 ns. **(B)** Interaction of lipopolysaccharides (LPS) with the hydrophobic pocket of MD2. “*” on TLR4 residues indicates chain B and “#” represents MD2 residues. **(C)** Interaction of N-terminal region of the leucine-rich repeat domain [leucine rich repeats (LRR)-NT; residues 24–51] and LRR1-3 with phospholipid headgroups. **(D)** An illustration of the hydrophobic cavity of MD2. **(E)** Volume of the MD2 hydrophobic cavity as a function of time. The horizontal white line indicates the average value. **(F)** Distance of the R2 chain terminal –CH_3_ group from the F126 of MD2 and F440 of TLR4. **(G)** Distance of LPS interacting atoms from K122 (MD2), K362 (TLR4), and S118 (MD2). **(H)** Root mean square deviation of LPS in both subunits of MD2 as a function of time. “*” indicates LPS in chain B of MD2.

During MD simulation, the horseshoe-like architecture of TLR4-ECD was intact in the membrane-aqueous environment with a leftward tilt due to electrostatic attraction between LRR-NT and DPPC headgroups (Figure 3A). Analysis of amino acid composition of the membrane-absorbed portion of ECD revealed the LRR-NT and LRR1-3 contain several polar and aromatic-polar residues (Figure 3C) that provide charge complementarity for association with the zwitterionic DPPC membrane surface. The electrostatic potential surface around TLR4 also showed that the membrane-absorbed portions of ECD comprised a zwitterionic patch as found on the DPPC surface (Figures 1E,F), supporting the charge-dependent tilting action of ECD on the bilayer.

Comparisons with the X-ray crystallographic structure (PDB ID: 3FXI) revealed that the dimerization interface between TLR4 and MD2 was completely conserved; this interface involved LRR15-17 of the C-terminal subdomain, as well as A and B patches provided by the N- and C-terminal regions of TLR4-ECD, respectively (12). TLR4 formed direct interactions with LPS and F126 (βG-βH strands) and the L87 loops (βE-βF strands) of MD2 (Figure 3B). The interaction of LPS within the β-cup-fold structure of MD2 is in agreement with the X-ray crystal structure. Specifically, all five acyl chains (R3, R2″, R2′, R3″, and R3′) of LPS are buried deep inside the MD2 pocket, while the R2 chain is partially exposed to the hydrophobic interface formed by F440, F463, and L444 of TLR4* and V82, M85, L87, I124, and F126 of MD2. The distances between the R2 chain terminal –CH_3_ group and the benzyl rings of F440 (TLR4*) and F126 (MD2) were consistent throughout the simulation (Figure 3F). The two phosphate groups of lipid A were anchored to a positively charged cluster of lysine and arginine from both TLR4 and MD2, providing dimerization support; specifically, K112 of MD2 and K362 of TLR4 were involved in a consistent hydrogen bond (H-bond) interaction with the phosphate oxygen atoms of lipid A throughout the MD simulation (Figure 3G). The phosphate oxygen of 1-PO_4_ forms a strong H-bond with the –OH group of S118 (Figure 3G). Overall, we observed that the effect of LPS binding and charge on the membrane led to localized changes in TLR4-ECD that allowed it to bend over the membrane surface at an approximate 45° angle.

Next, we monitored the volume of the MD2 large hydrophobic cavity to validate if membrane-bound TLR4-ECD possesses a physiologically relevant LPS-MD2 conformation. The LPS binding cavity of MD2 is highly flexible and can expand or shrink depending on the presence, absence, or size of the ligand. The LPS-bound MD2 cavity was reported to have a volume of ~1,710 Å^3^ (62). Our calculation revealed that the average volume of MD2 was approximately 1,700 Å^3^ value throughout the simulation (Figures 3D,E). This observation was supported by a stable RMSD of LPS as a function of simulation time (Figure 3H). Thus, MD2 maintains a steady interaction with LPS inside an intact cavity during the dynamic condition, where ECD tilts and adjusts itself on the membrane surface. Overall, we found the modeled TLR4 structure was reasonably accurate in mimicking the physiologically active state.

### TM Helices Tilt and Bend to Overcome Hydrophobic Mismatches

**Figure 4:**
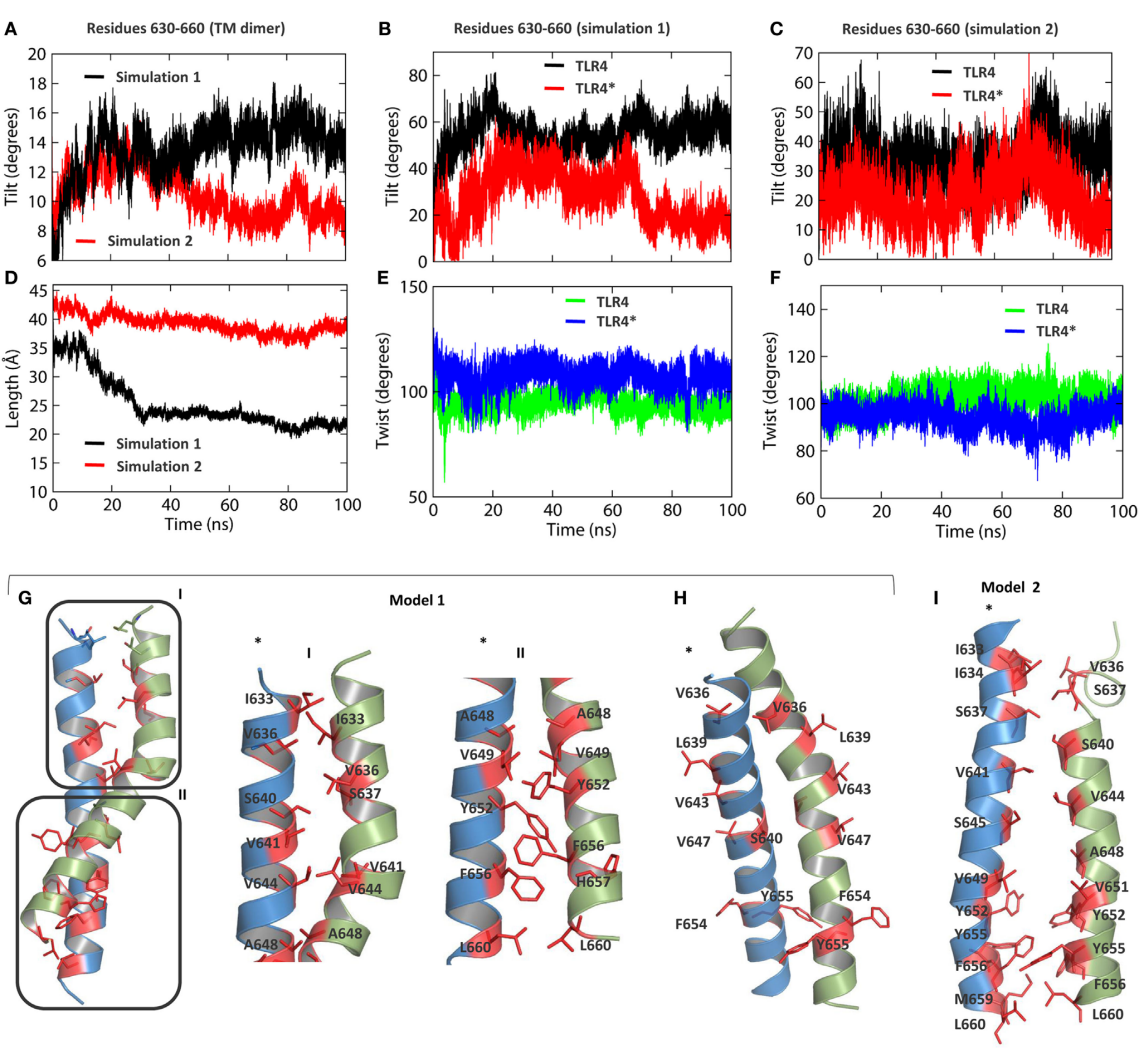
Helical properties of TLR4-transmembrane (TM). **(A)** Tilt angle of the TM dimer as a function of time. The black and red lines represent simulation 1 and simulation 2, respectively. **(B)** Tilt angles of individual helices in simulation 1. **(C)** Tilt angles of individual helices in simulation 2. **(D)** Length of the helical axis in both simulations. **(E)** Twist angle of TM residues in simulation 1. **(F)** Twist angle of TM residues in simulation 2. **(G)** Dimer packing interactions between residues of TM helices in simulation 1. **(H)** Solvent-exposed residues of the TM domain in simulation 1, indicating a possible alternate dimerization surface. **(I)** Dimer packing interactions between residues of TM helices in simulation 2.

We analyzed and compared the helical properties of TM bundles in two separate MD simulations. The TM domain in simulation 1 showed an axis-length of ~20 Å and a tilt angle of ~15° with respect to the average helical axis. In contrast, the TM domain in simulation 2 tilted between 8 and 10° with an axis-length of ~40 Å (Figures 4A,D). The shorter helical axis of the TM bundle in simulation 1 indicates a greater curvature than the TM bundle in simulation 2. The tilt angles of individual TM helices suggest that chain A largely contributes to the orientation of the whole TM domain (Figures 4B,C). During these tilting and bending processes, individual TM helices maintained a per-residue twist angle close to 100° (Figures 4E,F), a value usually obtained for ideal α-helices.

The dimer interface of TLR4-TM was studied in two different TLR4 models obtained from separate MD simulations. In model 1, the dimer interface can be divided into two regions, interface I with mostly aliphatic residues, namely I633, V636, V641, V644, and A648, and interface II containing both aliphatic and aromatic residues, namely V649, Y652, F656, and L660 (Figure 4G). Aromatic stacking was observed between Y652 and F656 of both monomers. S640 of interface I forms electrostatic interactions with backbone amide atoms of the other monomer residues. We found that residues V636, L639, V643, and V647—previously reported to form the dimer interface (31)—are exposed to the hydrophobic core of the bilayer (Figure 4H). This suggests the existence of an alternate dimer interface or a possible oligomerization interface of TLR4-TM, as reported for TLR3-TM (30). In model 2, we found some additional residues, namely I634, S637, and S645 of interface I and M659 of interface II, were involved in dimer formation (Figure 4I). However, the distance between the axis centers of the two helices in model 2 (~6.5 Å) was found to be larger than that of model 1 (~5 Å) (Figure S3A in Supplementary Material). This indicates that the tilt and curvature observed in model 1 indeed provides a stronger dimer packing between TM helices. Furthermore, we carried out a separate MD simulation of the isolated TM dimer (residues 630–660) by solvating it inside a DPPC membrane. Our analysis revealed that helical properties of the isolated TM dimer partly correlate with that of the full-length TLR4; specifically, the helical bundle tilts up to an angle of >40° with an axis-length of ~15 Å, much shorter than that of the full-length TLR4 (Figures S3D,F in Supplementary Material). While the twist angles remained close to 100° (Figure S3E in Supplementary Material), chain A contributed the most to the overall tilting behavior of the whole dimer (Figure S3C in Supplementary Material). However, distances between axis centers were found to be >10 Å (Figure S3B in Supplementary Material), indicating that isolated TMs tend to form a loose dimer in the membrane-bound condition.

### TIR Domains Are Partially Immersed Into the Lower Surface of the Membrane

**Figure 5:**
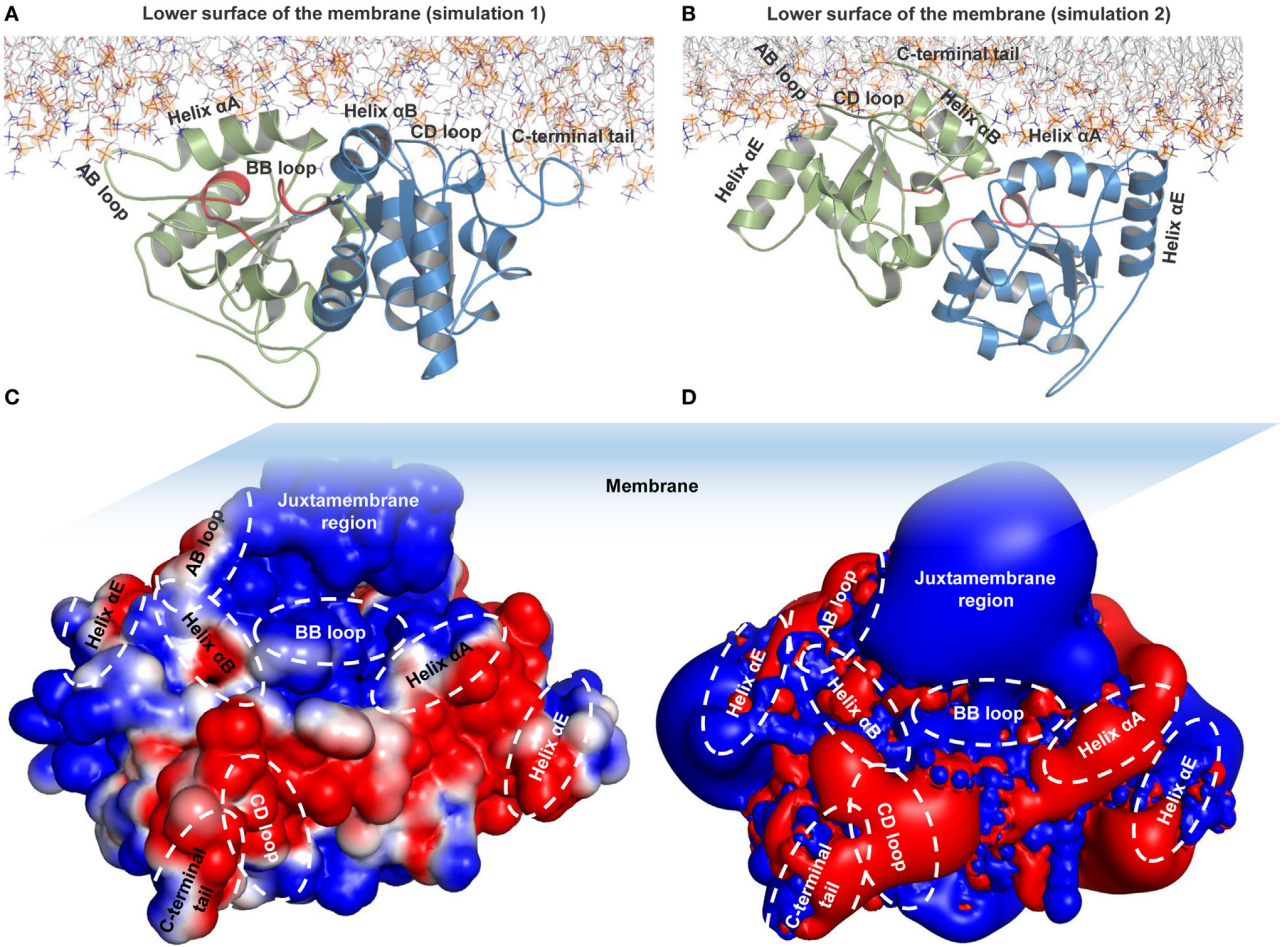
Membrane interaction and surface electrostatic properties of toll/interleukin-1 receptor (TIR) domains. **(A)** TIR-membrane interaction in simulation 1. **(B)** TIR-membrane interaction in simulation 2. **(C)** Electrostatic potential surface of the TIR domain in simulation 1. **(D)** Electrostatic isosurface showing only the most dominant positively and negatively charged surfaces of TIR domains.

During MD simulations, we observed that the TIR domains were partially absorbed into the lower leaflet of the bilayer, owing to electrostatic attraction by the polar headgroups supplemented by bending or tilting actions of both ECD and TM domains. The helix αA and AB loop of one subunit and the helix αB, CD loop, and C-terminal tail of the other subunit-mediated interactions with membrane phospholipids, while the BB loop was situated underneath the membrane (Figure 5A). The BB loop is considered the site of TIR dimerization and adaptor attachment (38), thus solvent exposure of this segment throughout MD simulation validates its functional significance. The DD loop, helix αE, and CD loops of TLR4-TIR were also situated adjacent to the membrane, but most residues from these segments remained directed toward the solvent. The C-terminal tail of one subunit is situated at the opposite end of the membrane closely spaced to helix αE. TIR domain residues that make direct contacts with phospholipids are shown in Table 2. The dimer interface mediated by the BB loop and helix αC of both monomers was found to be intact. Overall, the TIR dimer rotated up to 90° and moved upward before being absorbed into the membrane during MD simulation. All subdomains that contacted phospholipids in simulation 1 showed a similar behavior in simulation 2 (Figure 5B; Table 2). The electrostatic potential surface around the TIR domain indicated that the juxtamembrane region mostly contains positively charged residues; other surfaces bordering the membrane contain a mixture of positively and negatively charged patches that form polar contacts with the phosphate oxygen atoms of the DPPC headgroups (Figure 5C). Calculated electrostatic isosurfaces indicated that surfaces of the helix αA and CD loop were more negatively charged due to the presence of glutamic acids E685 and E691 in helix αA, and E750 and E752 in the CD loop (Figure 5D). Altogether, these analyses reveal that TLR4-TIR surfaces are potentially membrane-absorbed and solvent-exposed for interactions with other proteins.

**Table 2:** Amino acids of the TLR4-TIR domain that interact with the model phospholipid bilayer.

Simulation 1		Simulation 2	
Region	Residues	Region	Residues
Helix αA	Q683, E685, D686, W687, R689, N690, E691, K694	Helix αA*	D686, R689, N690, E691, K694, N695, E697
AB loop	E697, E698, V700, P701, Q704	AB loop	G699, V700, P701, P702, F703, Q704
Helix αB*	N721, H724, E725, H728, K729	Helix αB	A720, N721, H724, E725, H728, K729, S730
CD loop*	E750, Y751, E752, I753, A754, W757, Q758, F759, S762, R763	CD loop	A754, Q755, T756, W757, Q758, F757, S762, R763, A764
C-terminal tail*	T829, C831, N832, S838, I839	Helix αE	R809, R812, K813, L816, V800*, L802*, R804*, H805*, R812*
BB loop	Y9, R10	C-terminal tail	D817, K819, E824, C831, N832, W833, E835, A836, A837, S838, I839

*Indicates subunit B of TLR4.

### TAK-242 Binding Pockets Display Different Shapes in Isolated TIR and in Full-Length TLR4

**Figure 6:**
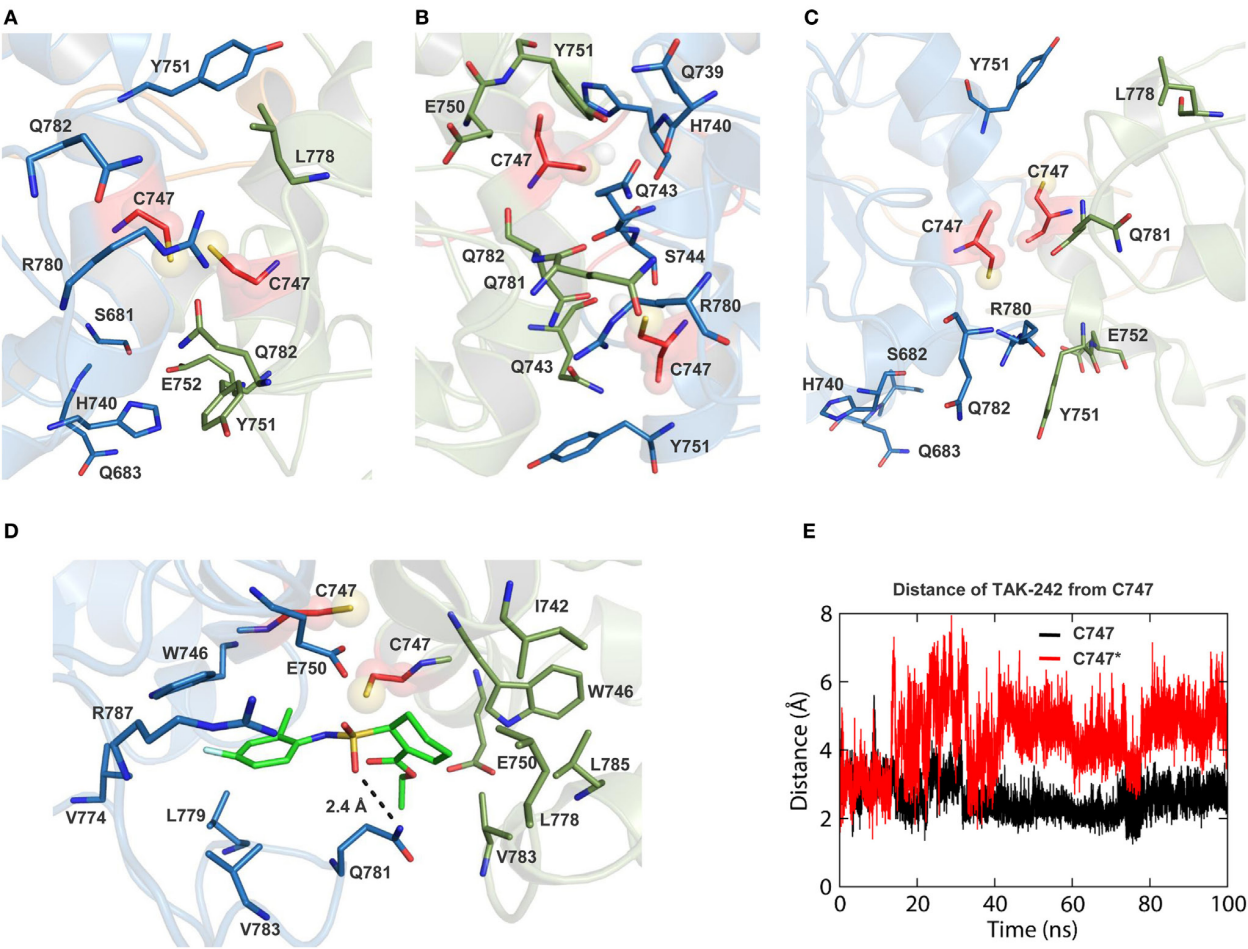
Views of TAK-242 binding cavities of different toll/interleukin-1 receptor (TIR) dimer models. **(A)** TAK-242 binding pocket of TIR dimer in simulation 1. **(B)** TAK-242 binding pocket of TIR dimer in simulation 2. **(C)** TAK-242 binding pocket of isolated TIR dimer. **(D)** Interaction of TAK-242 with the TIR domain dimer residues. **(E)** Distance between the TAK-242 cyclohexane ring and the –SH_2_ group of C747 of both TIR monomers.

TAK-242 is a well-known small molecular weight TLR4 antagonist that interacts with the amino acid, C747, situated in helix αC of the TIR domain. In our TLR10-TIR-based dimeric model, the BB loop and helix αC from one subunit form a dimerization interface with the corresponding segments from the other subunit, while C747 of each monomer faces each other creating a pocket for TAK-242 binding (Figures S4A,C in Supplementary Material) (63). We found that the opening of the TAK-242 binding cavity was lined with several bulkier amino acids, namely Y751, R780, L778, H740, and Q782, that partially blocked the cavity opening in both full-length TLR4 models (Figures 6A,B). However, in the isolated TIR dimer, these residues faced discrete directions providing a relatively exposed cavity for smoother ligand entry (Figure 6C). When a docked conformation TAK-242 inside an isolated TIR dimer was visualized after 100 ns of MD simulation, we found that the TAK-242 cyclohexane ring remained ~2.5 Å away from the –SH group of C747 throughout the simulation (Figure 6E). An H-bond was observed between the SO_2_-oxygen and nitrogen atoms of the Q781 side chain (Figure 6D). Interestingly, the residues surrounding C747 in unliganded TIRs did not interact with TAK-242, except for Q781. This suggests that TAK-242 induces conformational alterations in the residues neighboring C747 for its antagonistic interaction with TLR4. In an alternate TIR4-TIR dimeric model proposed by Toshchakov et al. (37), dimerization is governed by helix αE and the BB loop of either monomer (Figure S4B in Supplementary Material); this model shows that helix αC from both monomers is located at opposite surfaces of the TIR dimer with a solvent-exposed C747 (Figure S4D in Supplementary Material). Calculated binding affinities between TIR monomers revealed that αC-αC dimers were comparatively more stable than αE-BB dimers (Table 3). Furthermore, the binding affinity of TAK-242 for αC-αC dimers was found to be greater than that of TAK-242 for αE-BB dimers. Altogether, this indicates that the αC-αC orientation is the likely physiological dimeric state of TLR4-TIR domains.

**Table 3:** Comparison of binding affinities (kJ·mol^−1^) between different toll/interleukin-1 receptor (TIR) dimer models and TIR-TAK-242 complexes.

TIR interface	Δ_vdW_^a^	Δ_elec_^b^	Δ_ps_^c^	Δ_SASA_^d^	Δ_Total_^e^
Helix αC-αC	−437.952 (2.8)	−461.041 (3.6)	666.160 (5.4)	−62.136 (3.3)	−294.969 (4.6)
Helix αE-BB loop	−237.209 (1.8)	−581.003 (8.0)	665.111 (1.0)	−26.933 (4.6)	−180.035 (5.1)
TAK-242-αC-αC	−66.875 (2.3)	−72.790 (3.4)	101.001 (5.7)	−7.901 (1.9)	−46.566 (2.1)
TAK-242-αE-BB	−146.588 (1.4)	−181.268 (2.3)	328.178 (3.8)	−17.622 (1.0)	−17.299 (2.1)

*^a^Van der Waals energy*.

*^b^Electrostatic energy*.

*^c^Polar solvation energy*.

*^d^Solvent accessible surface area energy*.

*^e^Total binding free energy*.

*SD are indicated in brackets*.

## Discussion

An agonist-mediated dimeric state is the basic functional unit of TLRs; nevertheless, oligomeric states have lately been hypothesized (30, 64). Owing to a recent interest in TLR structural biology, NMR/X-ray crystal structures of almost all TLR individual domains have been solved. Although these experimental structures facilitated numerous mechanistic and rational drug development studies, the complete structural organization of ECD, TM, and TIR is yet to be studied as a single membrane-bound receptor unit (65). Here, we describe structural properties of a full-length TLR4 homo-heterodimer containing LPS-bound MD2 subunits in a membrane-aqueous environment that theoretically mimics the activated receptor in physiological conditions. Two independent MD simulations were performed and the most reasonable simulation showing stable receptor dynamics was described in detail. The rationale behind this is that the TM and TIR domains—with the exception of TLR4-ECDs—in simulation 2 displayed considerably dissimilar properties than those of simulation 1. We reason that due to a slightly different membrane placement of the TM bundle in simulation 2, the helical properties of the TM domain were partly distorted, causing the differential orientation of TIR domains. Therefore, we consider simulation 1 TLR4 as the most stable and acceptable model that describes various structural properties reliably. However, the observed tilt of TLR4 domains in simulation 1 might not be specific, since the juxtamembrane loops at the top and bottom layers of the membrane are highly flexible and mobile; therefore, the ECD and TIR domains can tilt to either direction of the membrane normal (*Z*-axis).

We found that the flexible juxtamembrane regions of full-length TLR4 allow for simultaneous tilting or bending actions of ECD and TIR domains on the membrane. As the ECD gradually inclined over the membrane surface, the LRR-NT of one monomer (TLR*) was partially absorbed into the upper leaflet of the bilayer. The inclination of ECD toward one particular direction of the bilayer surface was due to charge-dependent interactions of phospholipid headgroups with ECD amino acids facilitated by the cooperative actions of both TM and TIR domains. During this process, the LPS-bound MD2* (chain B of MD2) approached the membrane surface closely. It is possible that MD2 increased ECD bulkiness leading to its inclination over the membrane for stability. Interestingly, this dynamic behavior of ECD had little effect on the interaction between LPS and MD2. LPS was stable inside the large hydrophobic pocket of MD2—with an approximate volume of 1,700 Å^3^ (62)—and interacted consistently with both TLR4 and MD2. Earlier works have shown that the hexaacylated LPS tail prompts reorientation of the MD2 F126 aromatic side chain from an open to a closed conformation; this induces stable agonist binding prior to association with TLR4 (10, 66). The open conformation of F126 in agonist-free states of activated receptor dimers acts as a molecular switch that destabilizes the relative arrangement of TM and TIR domains (10). Throughout our simulation, F126 maintained a steady interaction distance with the LPS R2 chain that kept F126 in a closed conformation. This indicates that the observed dynamic properties of TLR4-ECD in the presence of the membrane bilayer do not destabilize interactions between different TLR4-MD2-LPS homo-heterodimer subunits.

TLR4, including all TLR members, has a typical type I membrane protein structure; a bulky ECD, a single narrow TM helix, and a small TIR domain. By observing this architecture, it is reasonable to accept that homo or heterodimerization is required to maintain stability at the TM region. This stability is important for the efficient adaptor recruitment platform provided by TIR domains in the cytoplasm. Studies on TLR-TM are sparse compared with ECD and TIR; however, the literature suggests that isolated TLR-TM segments can form stable homo/heterodimeric or oligomeric assemblies (30, 31, 64, 67). TLR4-TM (residues 630–650) along with the juxtamembrane region (residues 651–660) forms a continuous helix of 32 residues that extends beyond the nonpolar region of the membrane bilayer lower leaflet. This results in a hydrophobic mismatch between the nonpolar segments of the bilayer and the TM domain. Therefore, the TM domain tilts and bends considerably to overcome the energetic penalty incurred during dynamic conditions. More recently, the dimeric state of TLR4-TM was solved through an NMR spectroscopy study (31), showing a continuous helix that includes a portion of the ICD juxtamembrane region. Protein–protein docking was then applied to obtain a dimeric TM model that showed resides V636, L639, V643, and V647 from each monomer defining the potential dimer interface. Structures of TLR4 and TLR3-TM domains were determined by a synthetic TM construct that may not reflect the precise side chain orientations of a full-length TLR surrounded by membrane phospholipids. After a 100 ns-long MD simulation of full-length TLR4, we found that the residues reported by Mineev et al. (31) were directed toward the hydrophobic core of the bilayer; this points to the possibility of an alternate homodimerization or homooligomerization interface of TLR4-TM, as was found for other type I TM proteins, including TLR3-TM (30, 68). The strong interaction between aromatic residues of both TM monomers in interface II may result in helix bending at the center. These helical properties of TM domains of the full-length TLR4 are consistent with observations of other bitopic membrane proteins, where the specific lipid-TM and TM–TM interactions stabilize functionally relevant receptor conformations (69).

Inflammatory signaling and host defense downstream of membrane-bound TLRs involve several transient interactions between TIR domain-containing proteins (19). Functional TIR domain interactions are largely specific, in that a given set of TIRs tend to only associate with each other; however, some TIR domain-containing proteins interact with multiple partners giving rise to overlaps in the signal transduction cascade (22). Despite considerable efforts, the molecular basis of TIR domain specificities has not been completely unraveled. We observed that the TIR domains were gradually absorbed into the lower surface of the membrane bilayer due to electrostatic interactions and the bending or twisting actions of ECD and TM domains. Along with the juxtamembrane region, the upper surface of the TIR domain was partially immersed into the polar, lower face of the membrane. Of note, the primary contact surface between the membrane and both TIR domains is governed by helices αA and αB. Polar residues of the AB loop, CD loop, and C-terminal tail of one subunit made partial contacts with the membrane for stability, while the same regions of the other subunit remained completely solvent-exposed. The BB loop motif was situated right underneath the membrane bilayer as its Y709 and R710 residues formed H-bonds with phospholipid headgroups. The functionally important helix αE—thought to form an alternate dimerization surface by interacting with the BB loop (37)—remained solvent-exposed throughout the simulation, suggesting that helix αE might potentially form the TLR4 oligomerization interface. This indicates that our MD simulation accurately described physiological folding of membrane-bound TLR4. The interaction between TIR residues and membrane phospholipids or partial immersion of amino acid side chains into the polar region of the bilayer is unlikely to preclude these segments from contacting adaptor or other binding components. Decoy peptides from the helix αA and juxtamembrane region of TLR4-TIR were able to inhibit agonist-induced cytokine production by targeting their site of origin (37). This indicates that the polar region of the membrane, containing partially absorbed TIR residues, can accommodate other signaling components or external peptide antagonists. The MyD88 protein is sorted to TLR4-TIR by the membrane anchored adaptor, TIR domain-containing adaptor protein (TIRAP) (70, 71); therefore, it is most likely that the activated receptor complex formed by TLR4-TIRAP-MyD88 is in close proximity to membrane phospholipids (72). Moreover, it is likely that polar phospholipid headgroups provide the necessary charged environment for surface-exposed hydrophilic residues of the receptor or adaptors, thus stabilizing the supercomplex.

Next, we compared the small-molecule antagonist binding cavity of the TIR domain in both full-length TLR4 and isolated conditions. We considered TAK-242 as it is the most potent small-molecule antagonist reported to date that blocks recruitment of downstream adaptors by activated TLR4 (73). TAK-242 has been reported to bind to the conserved C747, located in the helix αC, of the TIR domain and prevents adaptor recruitment without affecting receptor homodimerization (63). In the absence of a commonly accepted TLR4-TIR dimerization model, we constructed two possible homodimerization interfaces, as reported in the literature. The first model was created based on the widely accepted view that helix αC and the BB loop of both subunits form the dimer interface (43, 44, 49). This model is based on the solved crystal structure of TLR10-TIR homodimer with one symmetric and one asymmetric subunits (28). We used the αC-αC model for completing our full-length TLR4 homodimer as well as for studying TAK-242 interactions. On the other hand, an alternative dimerization model has also been proposed using a decoy peptide approach (37). This model exposes helix αC toward the solvent and places helix αE and the BB loop in between the dimer interface. Our estimated binding free energy revealed that the αC-αC dimer has a greater binding affinity than the αE-BB dimer. Moreover, the affinity of TAK-242 for αC-αC dimers was stronger than for αE-BB dimers. This indicates that the αC-αC/BB-BB model might represent the physiological dimeric interface of TLR4. Remarkably, in the full-length TLR4, the TAK-242 binding cavity was partially blocked by neighboring residues that precluded C747 from contacting TAK-242. Possibly, due to the rotation and upward movement of the TIR dimer of full-length TLR4, the side chains of C747 neighboring residues covered the opening of the ligand binding cavity after coming in contact with rapidly moving water molecules. This phenomenon was also observed in a separate simulation of full-length TLR4. Thus, TAK-242 binding deep inside the TIR dimer cavity remains speculative, particularly in the absence of a cocrystallized ligand with the TIR dimer. Since TAK-242 does not interfere with LPS binding to MD2 and receptor dimerization, but prevents adaptor recruitment, it is tempting to state that C747 interacts with the ligand on the solvent accessible surface of the domain. An X-ray/NMR structure of TAK-242 bound to the dimeric TLR4-TIR is required to clarify this issue.

In this study, we proposed a full-length dimeric model of membrane-bound TLR4 containing two subunits of MD2-LPS complexes that represents the agonist-induced activated state. The structural properties of ECD, TM, and TIR domains of intact TLR4 are consistent with X-ray crystallography/NMR structures determined in isolated conditions. Lipid–protein and protein–protein interactions of the TM and TIR domains were crucial for shaping the biophysical properties behind the signaling-competent form of intact TLR4 homo-heterodimers (see Data Sheet 1 in Supplementary Material). Of note, caution should be exercised while using the proposed model in any study in the current form. Although we performed a detailed analysis and validation of its various structural properties in the membrane through multiple MD simulations, experiments with atomic force microscopy or cryo-electron microscopy on liposomes with inserted full-length TLR4-MD2-LPS complexes could only illustrate the exact conformation.

## Author Contributions

MP and HKK conceptualized the study. MP designed and performed the experiments. MP, HKK, and MB analyzed the results. MP, HKK, MB, and SC wrote the manuscript.

## Conflict of Interest Statement

The authors declare that the research was conducted in the absence of any commercial or financial relationships that could be construed as a potential conflict of interest.

## Footnotes

^1^http://www.ucalgary.ca/tieleman/.

^2^http://plasma-gate.weizmann.ac.il/Grace/.

^3^https://pymolwiki.org/index.php/Apbsplugin.

^4^http://nmr.chem.uu.nl/~adrien/course/molmod/get_timestamp.py.
