# Supplementary material for: Computational Insight Into the Structural Organization of Full-Length Toll-Like Receptor 4 Dimer in a Model Phospholipid Bilayer
Source: Front Immunol. 2018 Mar 12;9:489. doi: 10.3389/fimmu.2018.00489 (PMC5857566; doi:10.3389/fimmu.2018.00489)
Supplement: Supplementary file 2 [file Image_1.PDF]

Supplementary Material

Computational Insight Into the Structural Organization of Full-Length Toll-Like Receptor 4 Dimer in a Model Phospholipid Bilayer

Mahesh Chandra Patra[1], Hyuk-Kwon Kwon[2], Maria Batool[1], and Sangdun Choi[1*]

[1]Department of Molecular Science and Technology, Ajou University, Suwon, 16499, Korea

[2]Department of Orthopaedics and Rehabilitation, Yale School of Medicine, New Haven, CT 06510, USA

***Corresponding author**

Sangdun Choi, Professor

Department of Molecular Science and Technology

Ajou University, Suwon 16499, Korea

Phone: +82-31-219-2600

Fax: +82-31-219-1615

E-mail: sangdunchoi@ajou.ac.kr

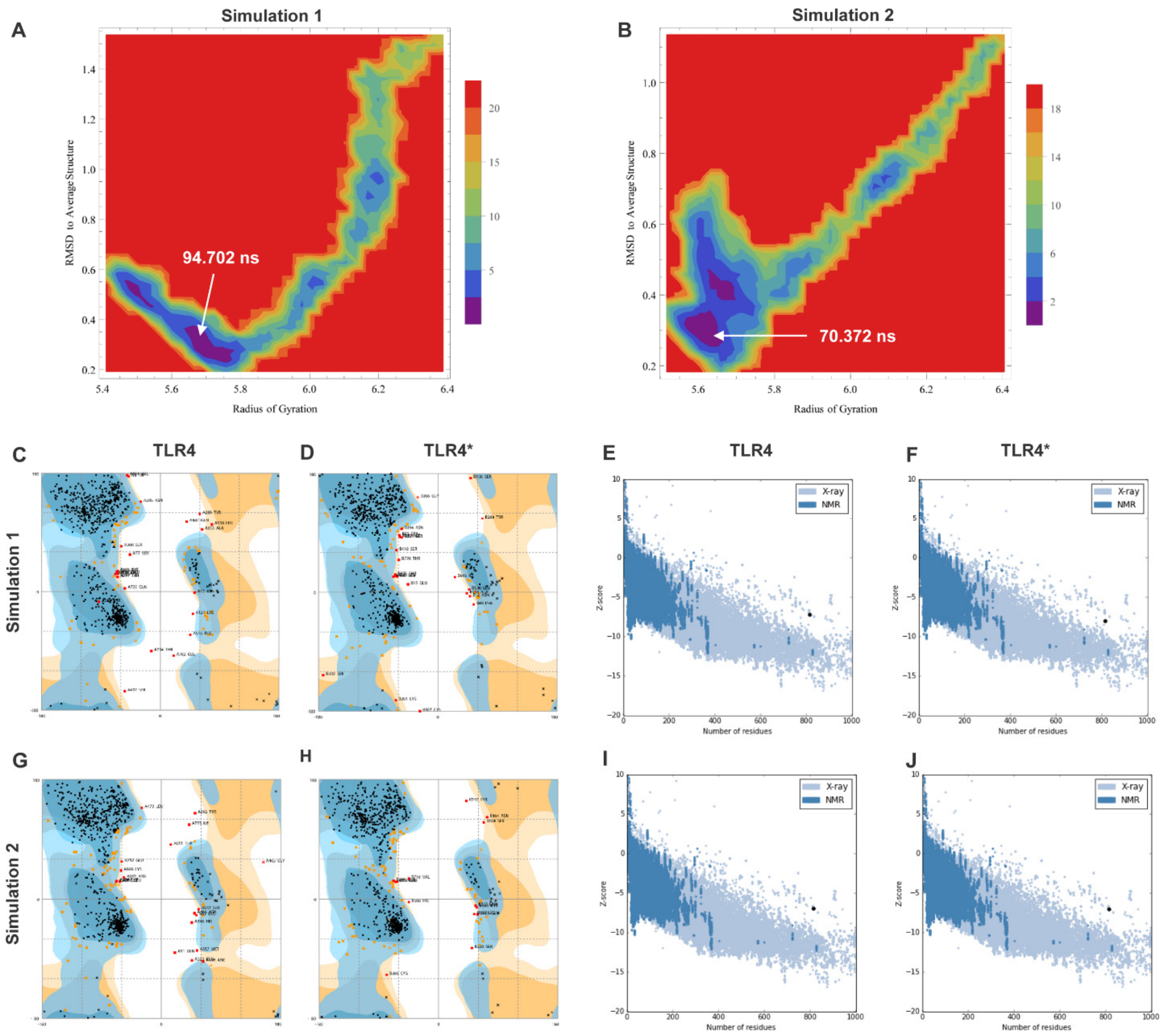

Figure S1. Model validation of TLR4 from two different MD simulations. Gibbs free energy landscapes of simulation 1 (**A**) and simulation 2 (**B**) showing low energy conformations concentrated at the blue region of the plot. The representative frames from 94.702 and 70.372 ns were used for model validation. Ramachandran plots of (**C**) TLR4 (chain A) and (**D**) TLR4* (chain B) of simulation 1. X- and Y-axes of the Ramachandran plot indicate phi (Φ) and psi (Ψ) dihedral angles, respectively. ProSA-web Z-scores for (**E**) TLR4 and (**F**) and TLR4* of simulation 1. Ramachandran plots of (**G**) TLR4 and (**H**) and TLR4* of simulation 2. ProSA-web Z-scores for (**I**) TLR4 and (**J**) and TLR4* of simulation 2.

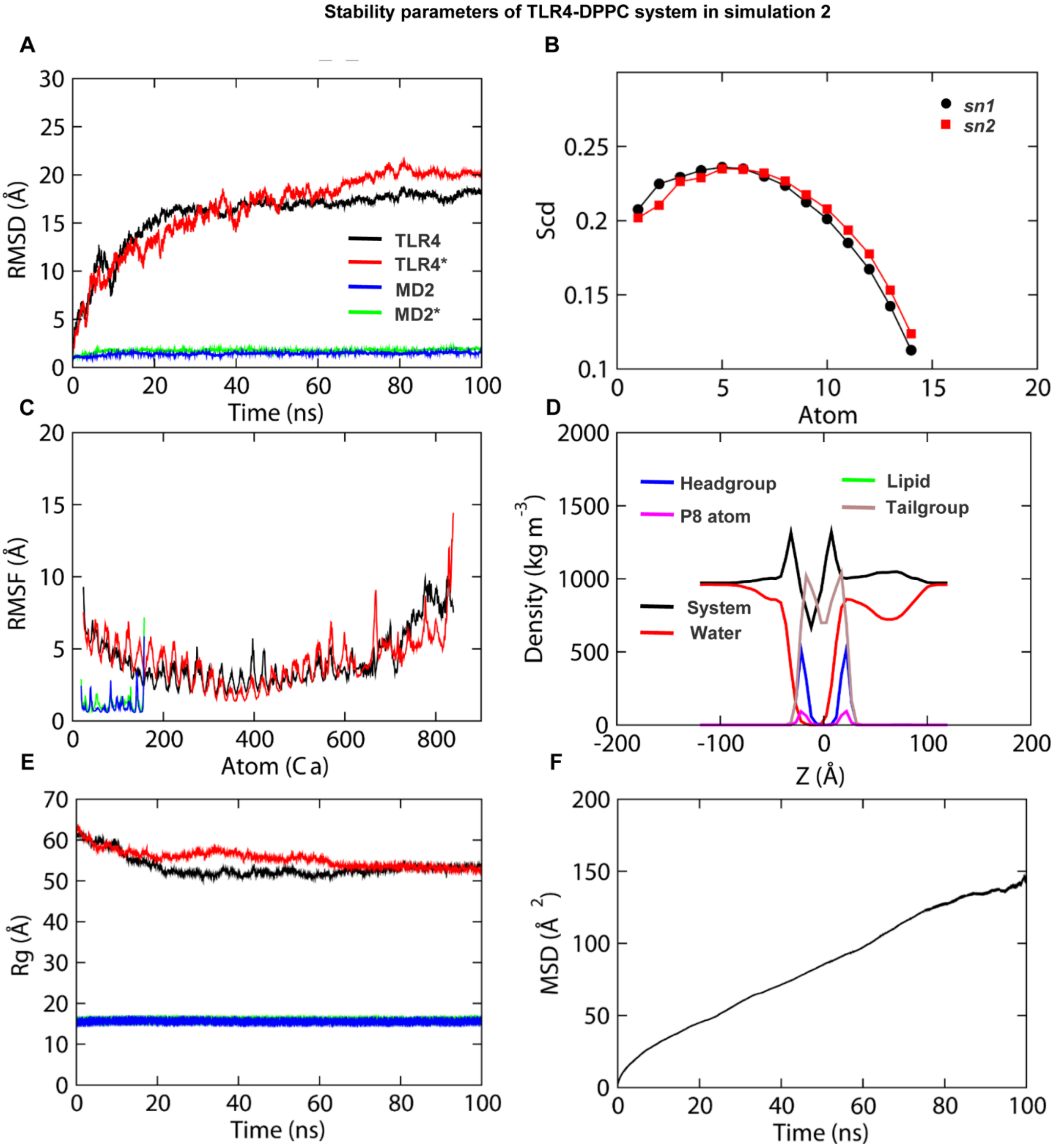

Figure S2. Stability parameters of the TLR4-MD2 complex and DPPC membrane of simulation 2 as a function of time. (**A**) Root mean square deviation. (**C**) Root mean square fluctuation. (**E**) Radius of gyration. (**B**) Order parameters for lipid acyl chains. (**D**) Density profiles of various components of the membrane. (**F**) Lateral diffusion of lipid headgroups, also known as the mean square displacement (MSD) of lipids. MSD values are based on diffusion of DPPC headgroup P8 atoms.

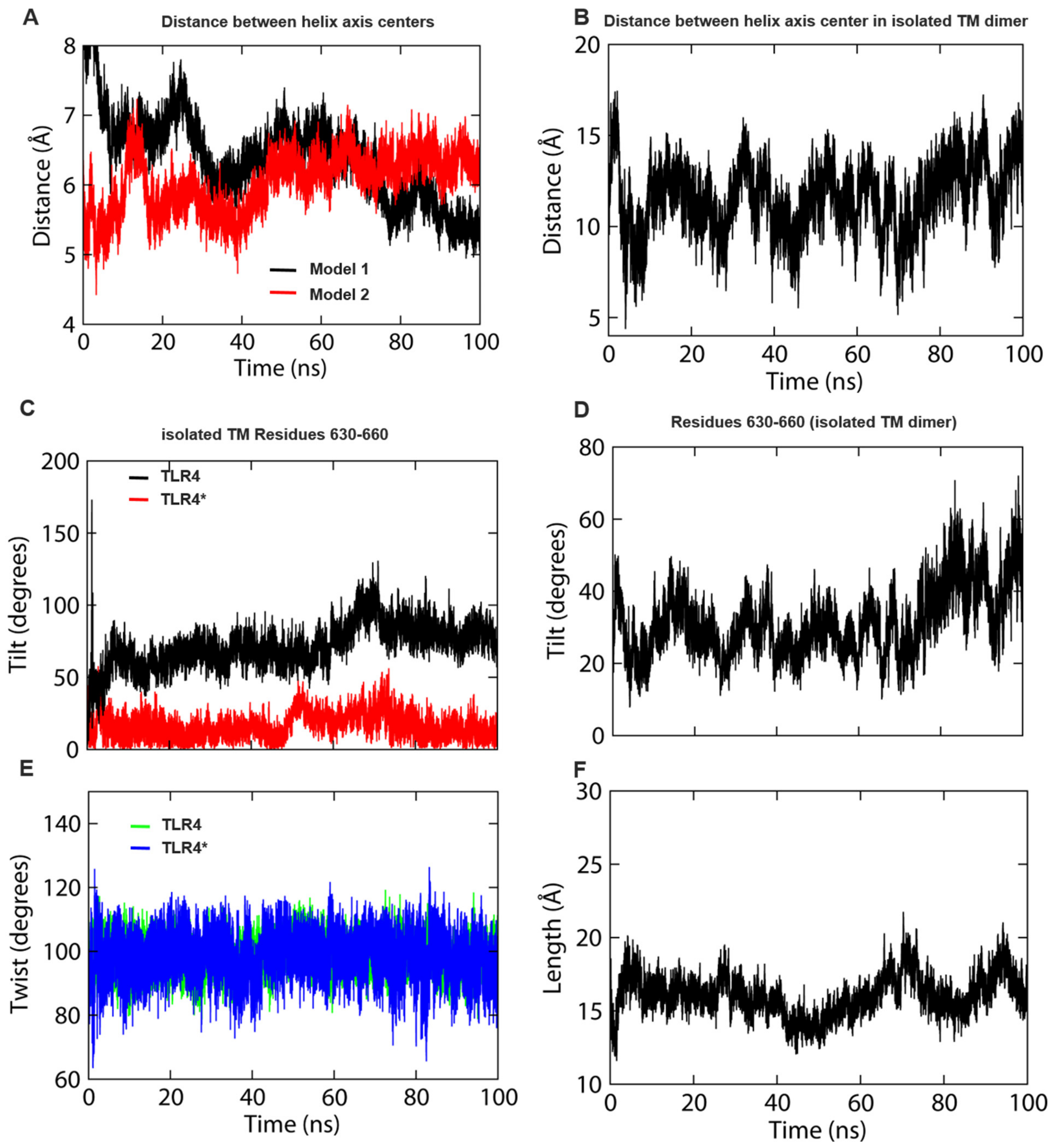

Figure S3. Helical properties of the simulation 2 TLR4-TM and the isolated TM dimer. (**A**) Distance between helices in model 1 and model 2 that correspond to simulation 1 and simulation 2, respectively. (**B**) Distance between helical axes in the isolated TLR4-TM dimer. (**C**) Tilt angles of TLR4-TM helices of simulation 2. Chain A is represented in black and chain B in red. (**D**) Helical tilt angle of the isolated TM bundle. (**E**) Twist angles of individual TLR4-TM helices of simulation 2. (**F**) Helical axis length of the isolated TLR4-TM bundle.

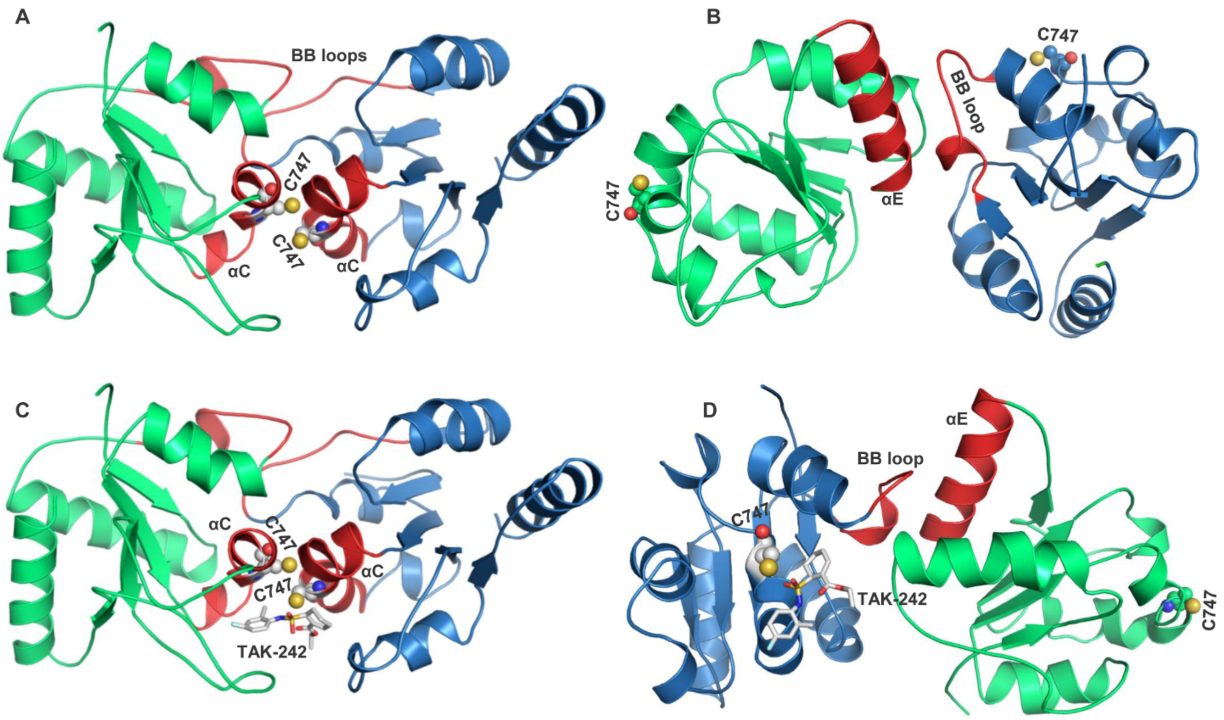

Figure S4. Two different dimeric models of TLR4-TIR. (**A**) TIR dimer based on an interface with αC and BB loops of both monomers. (**B**) TIR dimer based on an interface with αE of one monomer and the BB loop of the other. (**C**) Interaction of the TAK-242 ligand with C747 of the αC-αC dimer. (**D**) Interaction of TAK-242 with C747 of the αE-BB dimer. The dimer interfacing regions are marked in red.

TLR4.pdb. A computational model of the three dimensional structure of Toll-like receptor 4 dimer embedded in a dipalmitoylphosphatidylcholine membrane bilayer.
